# Supplementary material for: A Scoping Review of Food Literacy Interventions
Source: Nutrients. 2024 Sep 19;16(18):3171. doi: 10.3390/nu16183171 (PMC11435165; doi:10.3390/nu16183171)
Supplement: Supplementary file 1 [file nutrients-16-03171-s001.zip › nutrients-3172957-supplementary.pdf]

Database	Search terms				Additional Limits Applied
	Population	Exposure	Comparison	Outcome	
Medline	Limited to human studies	(Food literacy OR nutrition literacy OR food education OR nutrition education) AND (intervention)			Studies published in English
Cinahl		(Food Literacy OR nutrition literacy OR food education OR nutrition education) AND (intervention OR program)			Studies published in English
Amed		(Food literacy) OR (nutrition literacy) OR (food education) OR (nutrition education) AND (Intervention)			Studies published in English
Web of Science		(Food literacy OR nutrition literacy) AND (intervention)			Studies published in English
Proquest Education		(food literacy) OR (nutrition literacy) OR (food education) OR (nutrition education) AND intervention			Studies published in English

Table S1. Full search strategy for all databases.
